# Comprehensive analysis of lysine crotonylation in proteome of maintenance hemodialysis patients

**DOI:** 10.1097/MD.0000000000012035

**Published:** 2018-09-14

**Authors:** Wenbiao Chen, Donge Tang, Yong Xu, Yaoshuang Zou, Weiguo Sui, Yong Dai, Hongyan Diao

**Affiliations:** a State Key Laboratory for Diagnosis and Treatment of Infectious Diseases, Collaborative Innovation Center for Diagnosis and Treatment of Infectious Diseases, The First Affiliated Hospital, College of Medicine, Zhejiang University, Hangzhou; b Clinical Medical Research Center, the Second Clinical Medical College of Jinan University (Shenzhen People's Hospital), Shenzhen, Guangdong; c Nephrology Department of Guilin No.181 Hospital, Guangxi Key Laboratory of Metabolic Diseases Research, Guilin Key laboratory of Kidney Diseases Research, Guilin, Guangxi, P.R. China.

**Keywords:** lysine crotonylation, maintenance hemodialysis, mass spectrometry

## Abstract

**Introduction:**

Histone post-translational modifications (PTMs) carry epigenetic information to regulate diverse cellular processes at the chromatin level. Crotonylation, one of the most important and common PTMs, plays a key role in the regulation of various biological processes. However, no study has evaluated the role of lysine crotonylation in maintenance hemodialysis patients (MHP).

**Methods:**

Here, we comparatively evaluated the crotonylation proteome of normal controls (NC) and MHP using liquid chromatography tandem mass spectrometry (LC-MS/MS) coupled with highly sensitive immune-affinity purification.

**Results:**

A total of 1109 lysine modification sites distributed on 347 proteins were identified, including 93 and 252 crotonylated upregulated and downregulated proteins, respectively. Thus, a decrease in crotonylation of histone proteins was observed in patients with kidney failure undergoing maintenance hemodialysis. Intensive bioinformatic analysis revealed that most of the crotonylated proteins were distributed in the cytoplasm, nucleus, mitochondria, and extracellular region. Gene ontology enrichment analysis showed that the crotonylated proteins were significantly enriched in the platelet alpha granule lumen, platelet degranulation, and cell adhesion molecule binding. In addition, protein domain, including fibrinogen alpha/beta/gamma chain, zinc finger, and WD40-repeat-containing domain, were significantly enriched in crotonylated proteins. Kyoto Encyclopedia of Genes and Genomes (KEGG)-based functional enrichment analysis revealed that crotonylated proteins were enriched in complement and coagulation cascades, cardiac muscle contraction, and hematopoietic cell lineage, all of which have important associations with hemodialysis complications.

**Conclusions:**

This is the first report on the global crotonylation proteome of MHP. Lysine crotonylation was found to play important regulatory roles in pathophysiological processes in MHP.

## Abstract 2

Supplemental Digital Content is available in the text

## 1 Introduction

Protein post-translational modifications (PTMs) play essential roles in various cellular processes that modulates the physical and chemical properties, folding, conformation, stability, and activity of proteins, all of which may modify protein functions.^[1,2]^ The improved throughput of mass spectrometry (MS) technology has not only brought about a surge in proteome-scale studies but also identified several PTMs.^[3]^ Among all PTMs, lysine acetylation is one of the most studied PTMs. Protein lysine acetylation is a ubiquitous and reversible PTM first reported in histones that mainly regulated gene transcription and expression by the catalysis of histone acetyltransferases and histone deacetylases in the nucleus.^[4]^ The discovery of lysine acetylation on tubulin and mitochondrial proteins suggested an important role for lysine acetylation in cellular biology in addition to chromatin biology.^[5]^ Aside from lysine acetylation, some new types of PTMs, such as malonylation and lysine succinylation are identified as important regulatory mechanisms for a broad array of eukaryotic and prokaryotic physiological functions.^[6,7]^

Lysine crotonylation (Kcr) is a new protein PTM that was initially detected by Zhao's group in histones of human cell lines and mouse sperm.^[8]^ The discovery of Kcr has attracted attention of the scientific community and several researches have been directed to study Kcr. Kcr was first demonstrated as a robust indicator of active promoters and thought to be an important signal in the control of men germ cell differentiation.^[8]^ Sabari et al^[9]^ showed that histone acetyltransferase p300 possesses histone crotonyltransferase activity and demonstrated that p300-catalyzed histone crotonylation directly stimulates transcription to a level greater than that mediated by p300-catalyzed histone acetylation. Bao et al^[10]^ profiled “eraser” enzymes that recognize the lysine-4 crotonylated histone H3 (H3K4Cr) and found that Sirt1, Sirt2, and Sirt3 may catalyze the hydrolysis of lysine-crotonylated histone peptides and proteins. Moreover, Wei et al^[11]^ demonstrated that class I histone deacetylases (HDACs) are the major histone decrotonylases in mammals. Suberoylanilide hydroxamic acid (SAHA), a well-known HDAC, may inhibit decrotonylation on both histones and nonhistone proteins through the inhibition of HDACs.^[12]^ Li et al^[13]^ defined the evolutionarily conserved YEATS domain as a family of Kcr-favorable readers. Furthermore, Xiong et al^[14]^ demonstrated that histone acetylation-binding double PHD finger (DPF) domains of MOZ (KAT6A) and DPF2 (BAF45d) are engaged in a wide range of acylations at H3K14, with the highest affinity for crotonylation, which may act as a new regulatory mechanism of transcription control centered on the readout of histone Kcr. This discovery opened a new chapter in the regulation of Kcr. The study by Liu et al^[15]^ uncovered a biochemical pathway in the regulation of histone Kcr and implicated chromodomain Y-like protein-regulated histone Kcr in spermatogenesis. This study improved the understanding of the physiology of male reproduction and the mechanism underlying spermatogenic failure in azoospermia Factor c-deleted infertile men. Wei et al^[16]^ suggested that protein crotonylation may lead to the inhibition of DNA replication and, hence, influence cell cycle. Taken together, Kcr controls the interpretation of genetic information at the chromatin level and plays a key role in processes such as gene expression and cell fate.

Histone acetylation has been particularly well characterized and the purification and identification of histone acetyltransferases have improved our understanding of the role of targeted lysine acetylation in gene regulation.^[17,18]^ The most important role of histone acetylation is its involvement in gene regulation, and the aberrant regulation of histone acetylation is correlated with major human diseases.^[19]^ Until now, studies on histone acetylation have primarily focused on several diseases, including tumor,^[20]^ neuropsychiatric disorders,^[21]^ lupus,^[22]^ cardiovascular diseases,^[23]^ acute lymphoblastic leukemia,^[24]^ diabetic nephropathy,^[25]^ acute kidney injury, and chronic kidney disease (CKD).^[26]^ “The “writer” and “eraser” for this new histone modification have been reported based on the structural similarity between histone acetylation and histone crotonylation.”^[8,9]^ Furthermore, the same set of enzyme systems were shared by histone acetylation and histone crotonylation.^[9,11]^ Histone crotonylation is physiologically significant and functionally distinct from or redundant to histone acetylation.^[11]^ Hence, we speculate that Kcr exhibits a crucial role in a wide range of biological processes and may be critically implicated in the pathogenesis of diseases. However, very little is known on the involvement of Kcr in diseases and the underlying mechanism.

The prevalence of CKD and kidney failure (KF) is steadily increasing and becoming an important public health problem worldwide.^[27]^ The World Health Organization (WHO) report indicates that kidney diseases were responsible for 2,993,000 years of life lost and 38,104,000 disability-adjusted life years lost globally. Kidney transplantation is the best form of treatment. However, the rate of kidney transplant is very low, owing to the shortage of organ donors. Hemodialysis (HD) and peritoneal dialysis are the most common therapeutic options chosen by the patients when kidney transplantation is limited or contraindicated.^[28,29]^ It was verified that PTMs may form an epigenetic layer over the genetic layer that can respond to environmental cues and external stimuli to alter the expression of genes associated with CKD.^[26]^ The importance of epigenetic alterations, including PTMs, in fibrosis, inflammation, and immunity associated with various renal disorders and in kidney development is well appreciated.^[30]^ KF patients undergoing HD exhibit evident physiopathological changes in the internal environment, which could contributed to water and electrolyte balance disorder, resistance or hyperactivity of small molecules in vivo, organic lesions of various organs, and a decline in the immune system protection. A series of clinical symptoms could followed, such as hypertension or hypotension, hypokalemia or hypocalcemia, hyposecretion erythropoietin and hyperparathyroid parathormone, vascular fibrosis, and pigmentation of skin and so on. And then, HD complications, such as hemorrhage, uremic cardiomyopathy, stroke, anemia, poor nutrition, infection, and pneumonedema could happen and brought severe impairment in the quality of life and higher mortality to HD patients.^[31]^ Therefore, we speculate that Kcr has close relationship with the pathogenesis of KF and pathophysiological changes in patient undergoing maintenance HD.

Here, we investigated the global Kcr of proteome from normal control (NC) and maintenance hemodialysis patients (MHP) using high-resolution liquid chromatography tandem mass spectrometry (LC-MS/MS) coupled with highly sensitive immune-affinity purification. In total, we identified 1109 Kcr sites in 347 proteins. The identified crotonylated proteins were primarily localized in the cytoplasm, nucleus, mitochondria, and extracellular region. Bioinformatic analysis was performed to reveal the biological functions of crotonylated proteins; these proteins were significantly enriched in the platelet alpha granule lumen, platelet degranulation, and cell adhesion molecule binding and participated in complement and coagulation cascades, cardiac muscle contraction, and hematopoietic cell lineage pathways. To our knowledge, this is the first study to describe Kcr in the global proteome of MHP and expand our current understanding of Kcr roles in pathophysiological processes of KF patient undergoing maintenance HD.

## 2 Materials and methods

### 2.1 Samples collection

Peripheral blood used to validate candidate samples was collected from 16 patients with KF on maintenance HD and 16 NC. Patients were definitely diagnosed with KF and underwent HD for >6 months in Shenzhen People's Hospital. All 16 patients (age between 35 and 50) had chronic glomerulonephritis as baseline kidney disease and were receiving hemodialysis treatment 3 times a week. Moreover, all 16 patients had no obvious complications of hemodialysis for the time being. The peripheral blood of 16 patients were collected before the first hemodialysis in a week. The NC group (age between 30 and 50 years) corresponded to healthy subjects who presented to the hospital for routine health check-up and who had no kidney disease. All participants were free of clinical signs of any diseases, including cancer, allergic diseases, immune-compromised conditions, diabetes, and other infectious diseases. All patients and healthy controls provided written informed consent. The study was approved by the Clinical Research Ethics Committee of the Shenzhen People's Hospital. After sample collection, peripheral blood mononuclear cells (PBMCs) were isolated by density gradient centrifugation using Ficoll-Hypaque (GE Healthcare Bio-sciences AB, Uppsala, Sweden) according to the manufacturer's protocol. PBMCs were lysed with TRIzol reagent (Invitrogen, Carlsbad, CA) and stored at −80 °C.

### 2.2 Protein extraction

Sample was sonicated thrice on ice using a high intensity ultrasonic processor (Scientz) in a lysis buffer (8 M urea, 1% protease inhibitor cocktail). Cell debris were removed by centrifugation at 12,000 × *g* and 4 °C for 10 minutes. The supernatant was collected and protein concentration was determined with the bicinchoninic acid kit (Sigma Chemical Co., St. Louis, MO) according to the manufacturer's instructions.

### 2.3 Trypsin digestion

For digestion, the protein solution was reduced with 5 mM dithiothreitol for 30 minutes at 56 °C and alkylated with 11 mM iodoacetamide for 15 minutes at room temperature in the dark. The protein sample was diluted by adding 100 mM triethylammonium bicarbonate (TEAB) to urea concentration <2 M. The sample was overnight treated with trypsin at 1:50 trypsin-to-protein mass ratio for the first digestion, followed by the second digestion at 1:100 trypsin-to-protein mass ratio for 4 hours.

### 2.4 Tandem mass tag/isobaric tags for relative and absolute quantitation (TMT/iTRAQ) labeling

After trypsin digestion, the peptide was desalted with Strata X C18 SPE column (Phenomenex, Torrance, CA) and subjected to vacuum drying. The peptide was reconstituted in 0.5 M TEAB and processed according to the manufacturer's protocol for TMT kit/iTRAQ kit (AB Sciex, Foster City, CA). Briefly, 1 unit of TMT/iTRAQ reagent was thawed and reconstituted in acetonitrile. The peptide mixtures were incubated for 2 hours at room temperature and pooled, desalted, and dried by vacuum centrifugation.

### 2.5 High-performance liquid chromatography (HPLC) fractionation

The tryptic peptides were fractionated into fractions with high pH reverse-phase HPLC using Thermo Betasil C18 column (5 μm particles, 10 mm ID, 250 mm length). Briefly, peptides were first separated with a gradient of 8% to 32% acetonitrile (pH 9.0) over 60 minutes into 60 fractions. The peptides were then combined into 6 fractions and dried by vacuum centrifugation.

### 2.6 Affinity enrichment

To enrich Kcr-modified peptides, tryptic peptides dissolved in NETN buffer (100 mM sodium chloride [NaCl], 1 mM ethylenediaminetetraacetic acid [EDTA], 50 mM Tris–HCl, and 0.5% NP-40, pH 8.0) were incubated with pre-washed antibody beads (PTM Bio) at 4 °C overnight with gentle shaking. The beads were washed 4 times with NETN buffer and twice with water. The bound peptides were eluted from the beads with 0.1% trifluoroacetic acid. The eluted fractions were combined and vacuum dried. For LC-MS/MS analysis, the resulting peptides were desalted with C18 ZipTips (Millipore) according to the manufacturer's instructions.

### 2.7 LC-MS/MS analysis

The tryptic peptides were dissolved in 0.1% formic acid (solvent A) and directly loaded onto a home-made reversed-phase analytical column (15 cm length, 75 μm i.d.). The gradient comprised an increase from 6% to 23% of solvent B (0.1% formic acid in 98% acetonitrile) over 26 minute, 23% to 35% in 8 minute, and increasing to 80% in 3 minute, followed by a hold at 80% for the last 3 minute. All steps were performed at a constant flow rate of 400 nL/min on an EASY-nLC 1000 ultra-performance liquid chromatography (UPLC) system. The peptides were subjected to NSI source, followed by MS/MS in Q ExactiveTM Plus (Thermo) coupled online to UPLC. The electrospray voltage applied was 2.0 kV. The *m*/*z* scan range was 350 to 1800 for full scan and intact peptides were detected with an Orbitrap at a resolution of 70,000. Peptides were selected for MS/MS using NCE setting as 28 and the fragments were detected in the Orbitrap at a resolution of 17,500. A data-dependent procedure that alternated between one MS scan followed by 20 MS/MS scans with 15.0 second dynamic exclusion duration was used. Automatic gain control (AGC) was set at 5E4 and the fixed first mass was set as 100 *m*/*z*.

### 2.8 Database search

The resulting MS/MS data were processed using MaxQuant search engine (v.1.5.2.8). Tandem mass spectra were searched against the human database concatenated with a reverse decoy database. Trypsin/P was specified as the cleavage enzyme, allowing up to 4 missing cleavages. The mass tolerance for precursor ions was set as 20 ppm in the first search and 5 ppm in the main search, while that for the fragment ions was set as 0.02 Da. Carbamidomethylation on Cys was specified as fixed modification and oxidation on Met was specified as variable modification. False discovery rate (FDR) was adjusted to <1% and the minimum score for modified peptides was set as >40.

### 2.9 Gene ontology (GO) annotation

Gene ontology annotation was derived from the UniProt-GOA database (http://www.ebi.ac.uk/GOA/). The identified protein ID was converted to UniProt ID, followed by its mapping to GO IDs using the protein ID. If some identified proteins were not annotated by UniProt-GOA database, InterProScan software (http://www.ebi.ac.uk/interpro/search/sequence-search) was used to annotate protein's GO functions, based on protein sequence alignment method. The proteins were GO annotated based on the following 3 categories: biological process, cellular component, and molecular function. For each category, a two-tailed Fisher exact test was employed to test the enrichment of the differentially expressed protein against all identified proteins. GO with a corrected value of *P* < .05 was considered significant.

### 2.10 Domain annotation

The domain functional description of the identified proteins was annotated by InterProScan (a sequence analysis application) based on the protein sequence alignment method using InterPro (http://www.ebi.ac.uk/interpro/) domain database. For each category of proteins, InterPro database was searched and a two-tailed Fisher exact test was used to test the enrichment of the differentially expressed protein against all identified proteins. Protein domains with a corrected value of *P* < .05 were considered significant.

### 2.11 Kyoto encyclopedia of genes and genomes (KEGG) pathway annotation

We used KEGG online service tool KAAS (http://www.genome.jp/tools/kaas/) to annotate protein's KEGG database description. The mapping of the annotation result on the KEGG pathway database was performed using KEGG online service tool KEGG mapper. The pathway with a corrected value of *P* < .05 was considered significant. These pathways were classified into hierarchical categories according to KEGG website.

### 2.12 Subcellular localization

We used WoLF PSORT (http://www.genscript.com/wolf-psort.html), a subcellular localization predication software to predict subcellular localization.

### 2.13 Motif analysis

Soft motif-x (http://scan-x.med.harvard.edu/scan-x.html) was used to analyze the model of sequences comprising amino acids at specific positions of modify-21-mers (10 amino acids upstream and downstream of the site) in all protein sequences. All database protein sequences were used as the background database parameter, while other parameters were set as default.

## 3 Result

### 3.1 Comparative analysis of whole proteome and Kcr in MHP and NC

**Figure 1:**
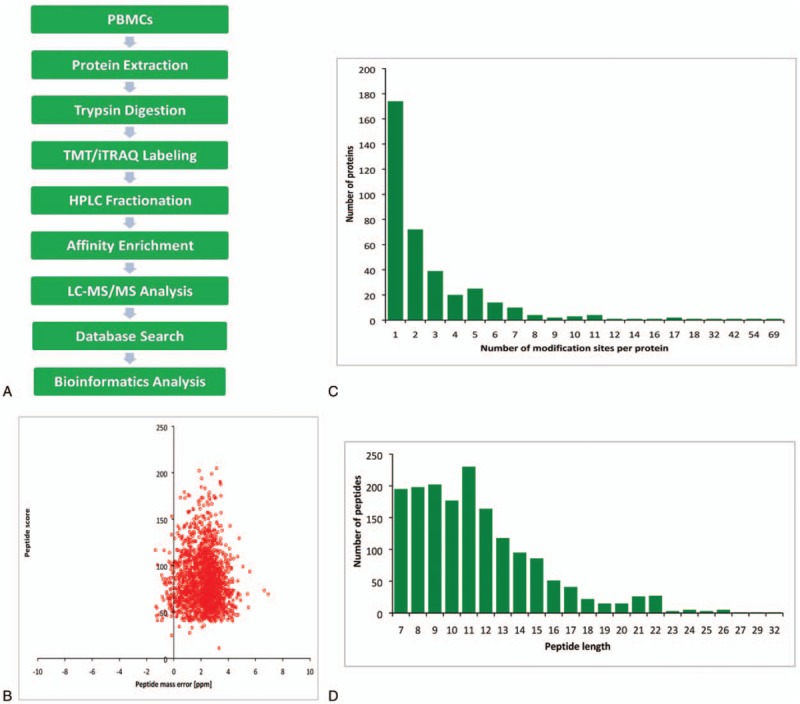
Comparative analysis of whole proteome and lysine crotonylation in MHP and NC. (A) Overview of experimental procedures used in the present study. (C) Distribution of lysine crotonylation in one protein. (D) Distribution of lysine crotonylation peptides based on their lengths. (B) Mass error distribution of all crotonylated peptides. MHP = maintenance hemodialysis patients, NC = normal controls.

The complete experimental procedure included 9 steps (Fig. 1A). Altogether, 1209 Kcr sites distributed in 377 proteins were identified and 1109 sites from 347 proteins were quantifiable (Table S1,). A fold change of >1.2 was considered as an upregulation, while that <1/1.2 was termed as downregulation. Differential expression in MHP and NC was observed at 860 sites for 345 proteins; these comprised 155 sites for 93 proteins that were upregulated and 705 sites for 252 proteins that were downregulated. All data are listed in Table S2. To confirm the validation of MS data, the mass error of all identified peptides was assessed. The mass error was centered around 0 and below 10 ppm, suggesting that the mass accuracy of MS data met the requirement (Fig. 1B). Among 347 crotonylated proteins, most proteins contained 1 or 2 crotonylation sites and few proteins had ≥7 or more crotonylation sites (Fig. 1C). Most peptides ranged from 8 to 20 amino acids in length, consistent with the rule of trypsin digestion (Fig. 1D).

### 3.2 Analysis of crotonylation site motif

**Figure 2:**
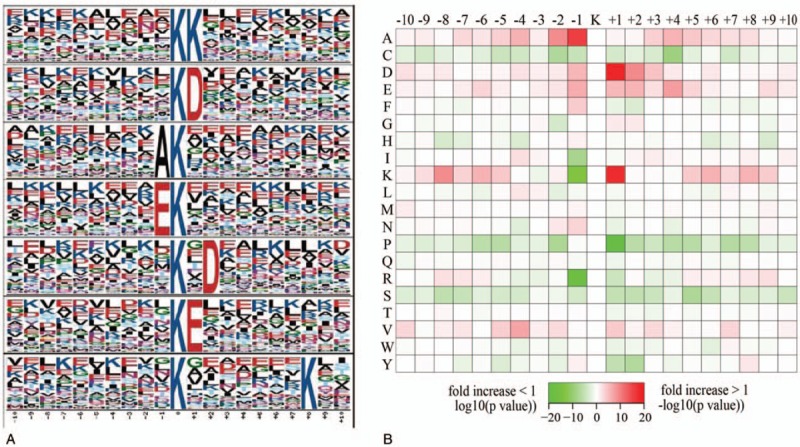
Analysis of crotonylation site motif. (A) Sequence probability logos of significantly enriched crotonylation site motifs around the lysine crotonylation sites. (B) Heat map of the amino acid composition around the lysine crotonylation sites showing the frequency of different types of amino acids around this residue. Red indicates enrichment and green indicates depletion.

To investigate the sequence commonalities surrounding crotonylation sites and compare them with acetylation sites, sequence motifs in all identified crotonylated peptides were investigated using the motif-X program. A total of 7 conserved motifs (KK, KD, AK, EK, K.D, KE, and K.......K) were retrieved (Table S3, Fig. 2A). In particular, motifs KE and KD were strikingly conserved. The significantly conserved amino acids in these motifs, namely E and D, were both negatively charged and rarely identified in other PTMs. These motifs are likely to represent a feature of crotonylation in MHP. In addition, hierarchical cluster analysis was performed to further analyze these motifs (Fig. 2B). The enrichment of positively charged K residues was observed at −10 to −5 and +10 to +5 positions, while negatively charged residues D and E were markedly enriched at −1 to +4 position. Short aliphatic A residues were frequently observed at −7 to +8 position, while the sulfur-containing C residue was undetected.

### 3.3 Functional classification of Kcr in GO and Kcr subcellular localization

**Figure 3:**
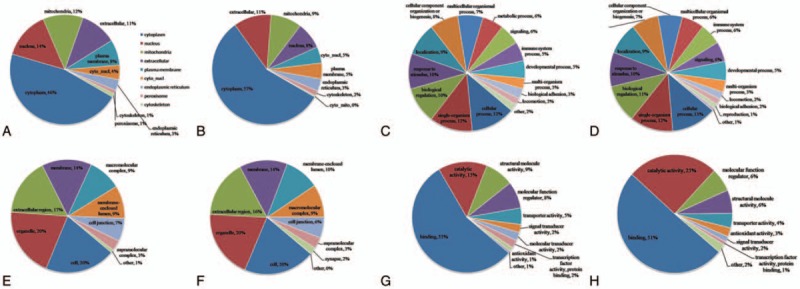
Functional classification of lysine crotonylation in GO analysis and subcellular localization of crotonylated proteins (A) and (B) show subcellular localization of upregulated and downregulated crotonylated proteins, respectively. (C) and (D) show results of GO analysis for upregulated and downregulated crotonylated proteins in biological processes, respectively. (E) and (F) show results of GO analysis for upregulated and downregulated crotonylated proteins in cellular component, respectively. (G) and (H) show results of GO analysis for upregulated and downregulated crotonylated proteins in molecular function, respectively. GO = gene ontology.

The subcellular localization of the identified Kcr was characterized (Fig. 3A), and the majority of upregulated crotonylated proteins was distributed in the cytoplasm (46%), nucleus (14%), mitochondria (12%), and extracellular region (11%). In contrast, the majority of the downregulated proteins was distributed in the cytoplasm (57%), extracellular region (11%), mitochondria (9%), and nucleus (8%) (Fig. 3B). According to the data, a significant difference was not observed in the localization of the upregulated and downregulated crotonylated proteins.

To obtain an overview of crotonylated proteins in MHP, GO functional classification was evaluated for all crotonylated proteins based on their biological processes, molecular functions, and cellular components (Table S4). Within the biological processes category, most of the upregulated crotonylated proteins were related to cellular process, single-organism process, biological regulation, and response to stimulus (Fig. 3C). In contrast, most of the downregulated proteins were related to cellular process, single-organism process, biological regulation, and response to stimulus (Fig. 3D). Within the cellular component category, most of the upregulated crotonylated proteins were related to cell, organelle, extracellular region, and membrane (Fig. 3E), while most of the downregulated proteins were related to cell, organelle, extracellular region, and membrane (Fig. 3F). Within the molecular function category, most of the upregulated crotonylated proteins were related to binding, catalytic activity, structural molecule activity, and molecular function regulator (Fig. 3G), while most of the downregulated proteins were related to binding, catalytic activity, molecular function regulator, and structural molecule activity (Fig. 3H). GO functional classification revealed no significant difference between the upregulated and downregulated crotonylated proteins and suggested that Kcr may have extensive biological functions.

### 3.4 Functional enrichment of Kcr in GO, KEGG, and protein domain

**Figure 4:**
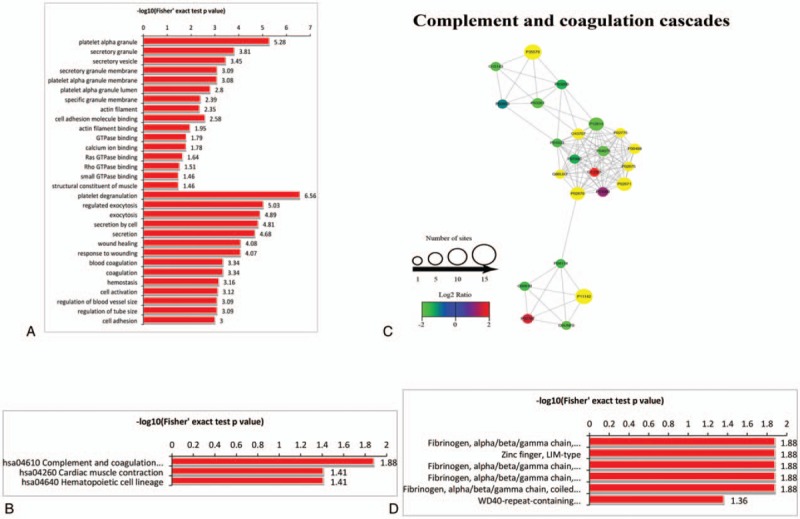
Functional enrichment of lysine crotonylation in GO, KEGG, and protein domain. (A) GO-based enrichment analysis of upregulated crotonylated proteins in terms of cellular component, molecular function, and biological process. (B) KEGG-based enrichment analysis of upregulated crotonylated proteins. (C) Protein–protein interaction network of complement and coagulation cascades pathway. (D) Protein domain-based enrichment analysis of upregulated crotonylated proteins. GO = gene ontology, KEGG = Kyoto Encyclopedia of Genes and Genomes.

The result of GO-based functional enrichment analysis is shown in Table S5. The upregulated crotonylated proteins were highly enriched in the cellular component of platelet alpha granule, secretory granule, and secretory vesicle and molecular functions, including cell adhesion molecule binding and actin filament binding, and biological processes, including platelet degranulation, regulated exocytosis, and exocytosis. However, no downregulated crotonylated proteins could be enriched in GO analysis (Fig. 4A). KEGG-based functional enrichment analysis (Table S6) showed that the upregulated crotonylated proteins were only enriched in 3 important processes, including complement and coagulation cascades, cardiac muscle contraction, and hematopoietic cell lineage, all of which had important relationship with HD complications (Fig. 4B). Crotonylated protein involved in complement and coagulation cascades were retrieved, comprising a dense protein interaction network (Fig. 4C). However, no downregulated crotonylated proteins could be enriched in KEGG analysis. Furthermore, upregulated crotonylated protein domains such as fibrinogen, alpha/beta/gamma chain, C-terminal globular, subdomain 2; zinc finger, LIM-type; fibrinogen, alpha/beta/gamma chain, C-terminal globular domain; fibrinogen, alpha/beta/gamma chain, C-terminal globular, subdomain 1; fibrinogen, alpha/beta/gamma chain, coiled coil domain; and WD40-repeat-containing domain were enriched (Table S7, Fig. 4D), suggestive of the important role of Kcr in these processes. No downregulated crotonylated proteins were observed in the protein domain.

### 3.5 Cluster analyses in GO, KEGG, and protein domain

**Figure 5:**
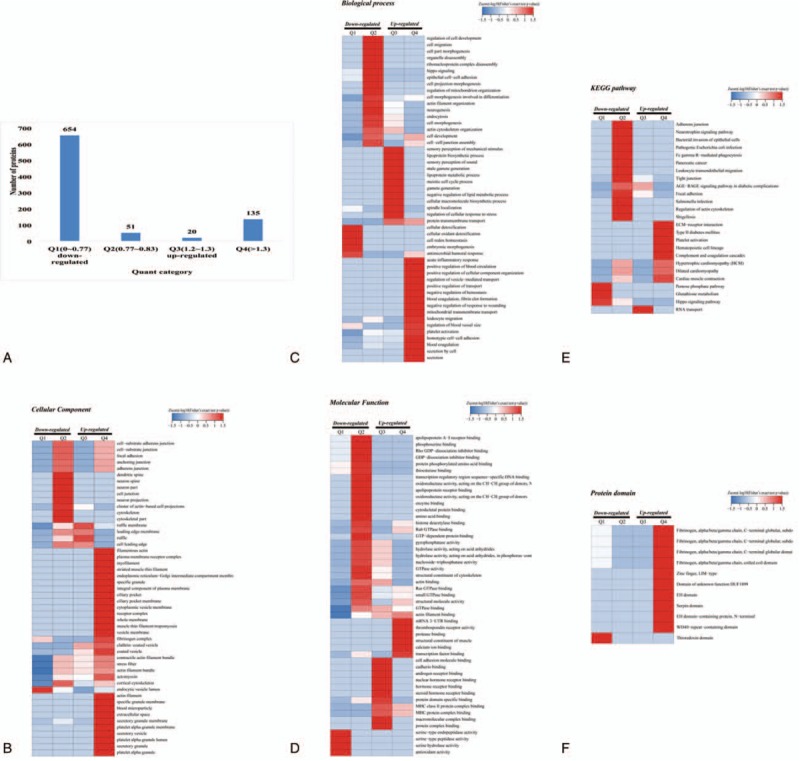
Cluster analysis in GO, KEGG, and protein domain. (A) Quantified crotonylated proteins were divided into 4 quantiles. (B) Cellular component analysis. (C) Biological process analysis. (D) Molecular function analysis. (E) KEGG pathway analysis. (F) Protein domain. GO = gene ontology, KEGG = Kyoto Encyclopedia of Genes and Genomes.

To evaluate further details of the functions of Kcr, cluster analyses based on GO, KEGG, and protein domain enrichment were performed. All quantified crotonylated proteins were divided into the following 4 quantiles (Q1–Q4) according to fold changes of Kcr sites: Q1 (0 < ratio < 0.77), Q2 (0.77 < ratio < 0.77), Q3 (1.2 < ratio < 1.3), and Q4 (ratio > 1.3) and *P* value <.05. We identified 381, 80, 57, and 252 crotonylated proteins in Q1, Q2, Q3, and Q4, respectively. The quantifiable proteins from the 4 categories were plotted for the enrichment-based cluster analysis. Q1 and Q2 were regarded as downregulated proteins, while Q3 and Q4 were regarded as upregulated proteins (Fig. 5A).

For GO analysis, crotonylated proteins in Q2 were localized on the cell substrate, junction, neuron, and cytoskeleton, while the crotonylated proteins in Q4 were mainly enriched in cellular secretory, cellular membrane, and cellular microstructure in the cellular component (Fig. 5B). Biological process enrichment of crotonylation was performed and crotonylated proteins in Q2 were found to be localized in cellular functions such as migration and development. Crotonylated proteins in Q3 were mainly enriched in cellular metabolic process and cellular response to outside. Moreover, crotonylated proteins in Q4 were mainly enriched in cellular response to the body pathophysiology (Fig. 5C). Crotonylated proteins in molecular function were highly enriched in the cellular binding process and cellular activity of enzymes in Q1, Q2, Q3, and Q4 (Fig. 5D).

KEGG enrichment of the crotonylated proteins revealed some pathways that were related to diseases in Q2 and Q4, such as pancreatic cancer, type II diabetes mellitus, dilated cardiomyopathy, and *Salmonella* infection, while Q1 crotonylated proteins were enriched in cellular signaling pathway and cellular metabolism (Fig. 5E).

Crotonylated proteins of protein domain were analyzed. These were highly enriched in Q4 with fibrinogen, alpha/beta/gamma chain, C-terminal globular, subdomain 2; zinc finger, LIM-type; fibrinogen, alpha/beta/gamma chain, C-terminal globular domain; fibrinogen, alpha/beta/gamma chain, C-terminal globular, subdomain 1; fibrinogen, alpha/beta/gamma chain, coiled coil domain; and WD40-repeat-containing domain, similar to the functional classification of protein domain (Fig. 5F).

### 3.6 Protein–protein interaction network of the Kcr proteins

**Figure 6:**
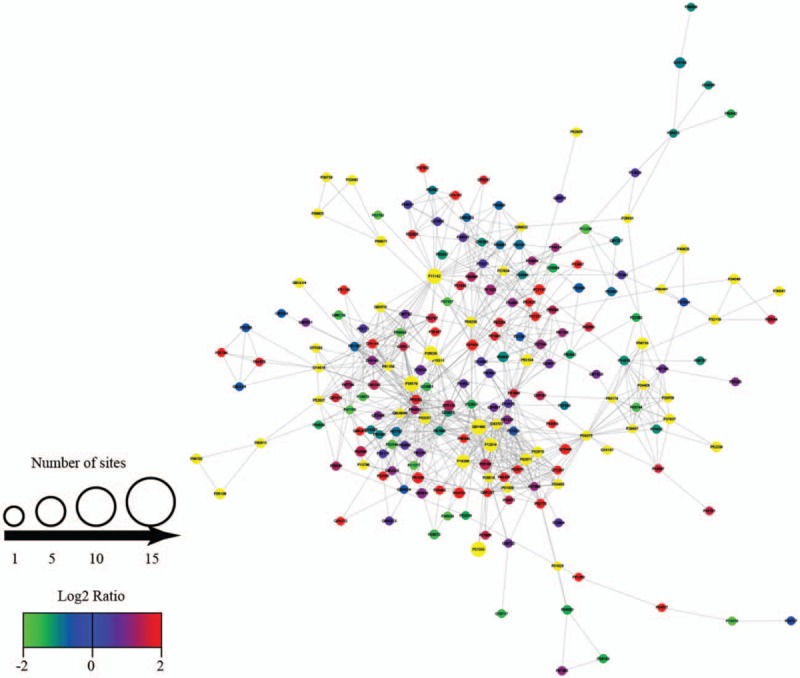
Protein–protein interaction network of the Kcr proteins. Kcr = lysine crotonylation.

To further identify the cellular processes regulated through crotonylation in MHP, protein−protein interaction network of the Kcr proteins was established (Fig. 6). A total of 888 protein–protein pairs mapped to the protein interaction database, presenting a global view of the diverse cellular functions of crotonylated proteins in MHP. The physiological interactions among these crotonylated protein complexes likely contribute to their cooperation and coordination in MHP.

## 4 Discussion

To the best of our knowledge, this is the first study to describe Kcr in KF patients on maintenance HD. Several evidences support the role of variations in PTMs in KF. A recent study has associated key events related to KF such as dysregulated expression of genes related to fibrosis, cell cycle, and inflammation with changes in PTMs.^[26]^ Sun et al^[32]^ found that transferrin growth factor (TGF)-β upregulated the expression of H3K4-methyltransferase SET7, which enhanced the expression of TGF-β-induced fibrotic genes. These events may contribute to renal fibrosis changes in KF, implicating the key role of histone methylation in KF. During histone acetylation, TGF-β promotes p300 recruitment with a concomitant increase in H3K9/14Ac and chromatin relaxation at fibrotic gene promoters and Smad2/3 acetylation/activation, leading to an increase in fibrosis in diabetic renal failure.^[33]^ We found an increase in the expression of HDAC-2, -4, and -5 in kidney biopsies from patients with type 1 diabetes. HDAC4-signal transducer and activator of transcription 1 (STAT1) signaling promotes podocyte injury, leading to CKD or KF.^[34]^ Although PTMs have been studied in a variety of model systems, the biochemical function of Kcr in KF patients on maintenance HD is poorly understood.

In this study, we comparatively evaluated the crotonylation proteome of both NC and MHP and identified crotonylated peptides by antibody affinity enrichment, followed by high-resolution mass spectrometry. This approach facilitated the identification of 1109 sites from 347 proteins. Among the quantitative Kcr data set, 155 and 705 sites, corresponding to 93 and 252 proteins were significantly upregulated and downregulated, respectively. According to above data, Kcr levels in histone proteins decreased in MHP compared with NC. Kidney tissue histone crotonylation was shown to increase during acute kidney injury. The elevated histone crotonylation may have a beneficial effect on acute kidney injury, as evident from the increase in the level of peroxisome proliferator-activated receptor gamma coactivator 1-alpha (PGC-1α) expression in cultured tubular cells and healthy kidneys.^[35]^ PGC-1α, a major regulator of mitochondrial biogenesis and metabolism, not only suppresses the degree of renal impairment but also helps in the recovery from this disorder.^[36]^ Acute kidney injury is a cause of chronic KF. However, acute kidney injury may be reversed by early treatment to prevent chronic KF. Histone crotonylation may be helpful for the recovery from acute kidney injury, as evident from the increase in the level of histone crotonylation during acute kidney injury. However, it was impossible to restore kidney function in patients on maintenance HD. This observation explains why Kcr levels were lower in histone proteins from MHP as compared with NC.

Functional enrichment of Kcr in GO analysis revealed the crotonylated proteins were associated with diverse biological processes, including cellular structure components, cellular molecular binding, and pathophysiological processes of cellular involvement. Thus, a wide range of interactions involved in these biological processes may be modulated through protein crotonylation. The functional enrichment of Kcr in KEGG analysis revealed only 3 pathways, including complement and coagulation cascades, cardiac muscle contraction, and hematopoietic cell lineage, all of which have important relationship with HD complications. The activation of complement and coagulation cascades system is known to be common during extracorporeal procedures such as HD, wherein the human blood is exposed to biomaterial surfaces.^[37]^ HD is associated with complement and coagulation cascades reactions that cause whole body inflammation and may contribute to accelerated arteriosclerosis, ultimately leading to cardiovascular events.^[38]^ Patients undergoing maintenance HD develop both structural and functional cardiovascular abnormalities.^[39]^ Despite improvement in the dialysis technology, cardiovascular mortality in this population is high. A large proportion of patients on HD dying of cardiac issues fail to show cardiac muscle contraction.^[40]^ Postmortem and biopsy studies have demonstrated that patients with CKD and end-stage renal disease on HD have high levels of interstitial myocardial fibrosis, which was the main reason for the absence of cardiac muscle contraction.^[41]^ Furthermore, HD influences the cardiovascular system and results in hemodynamic disturbances as well as electrolyte shifts, which alter the myocardial electrophysiology. The loss of hematopoietic cell lineage was a commonly diagnosed complication among MHP. Several factors such as the deterioration of kidney function, iron deficiency, poor nutrition, and uremic toxins are known to impair the function of hematopoietic stem cells.^[42]^ Therefore, additional treatment with erythropoietin that drives the bone marrow to produce blood is essential for MHP. At the same time, these patients need HD (for the removal of toxins) and iron supplements as well as highly nutritive substances as the raw material for blood cell.^[43]^ Our study results indicate the possible involvement of Kcr in HD complications. Kcr may reduce HD complications; further research is needed to reveal the role of Kcr in the global proteome of patients on maintenance HD.

## Acknowledgments

The authors thank maintenance hemodialysis patients and healthy persons for participating in the basic research of this project.

## Author contributions

**Conceptualization:** Donge Tang.

**Data curation:** Donge Tang.

**Funding acquisition:** Dai Yong.

**Investigation:** Weiguo Sui.

**Methodology:** Yong Xu.

**Project administration:** Aoshuang Zou, Hongyan Diao.

**Resources:** Aoshuang Zou.

**Supervision:** Dai Yong, Weiguo Sui, Hongyan Diao.

**Visualization:** Yong Xu.

**Writing – original draft:** Wenbiao Chen.

**Writing – review & editing:** Wenbiao Chen.

## Supplementary Material

Supplemental Digital Content

## Supplementary Material

Supplemental Digital Content

## Supplementary Material

Supplemental Digital Content

## Supplementary Material

Supplemental Digital Content

## Supplementary Material

Supplemental Digital Content

## Supplementary Material

Supplemental Digital Content

## Supplementary Material

Supplemental Digital Content
